# An interventional model to develop health professionals in West Africa

**DOI:** 10.11604/pamj.2014.18.152.3283

**Published:** 2014-06-17

**Authors:** Anselme Simeon Sanou, Florence Adeola Awoyale, Abdoulaye Diallo

**Affiliations:** 1 Centre MURAZ Research Institute, Bobo-Dioulasso, Burkina Faso; 2 West African Health Organization, Headquarters, Bobo-Dioulasso, Burkina Faso

**Keywords:** Interventional model, human resource, West African Health Organization, health professional

## Abstract

The health sector is characterized by a human resource base lacking in numbers, specialized skills, and management skills. West African Health Organization (WAHO) recognizes the need within the West Africa sub-region for bilingual professionals who are skilled in public health, management, leadership, and information technology to build human capacity in public health and developed the Young Professionals Internship Program (YPIP). Our study explores the evolution of the programme. YPIP program has successfully carried out its original aims and objectives to equip young professionals with basic principles of public health, management, and leadership, acquire competence in a second official language (French, English, and Portuguese), information and communication technology. Contributing factors towards this successful evaluation included positive ratings and commentary from previous interns about the relevance, usefulness, and quality of the programme, encouraging feedback from WAHO management, trainers, administrators, and intern employers on the impact of the YPIP program on young professionals, supporting evidence that demonstrates increased knowledge in professional skills and language competency.

## Introduction

The health sector is characterized by a human resource base lacking in numbers, specialized skills, management skills, barriers such as language. Technical competences among human resources are inadequate to meet national health targets and global health goals. Human resources play a critical role in delivering health services to the population. Health planners and decision-makers have to ensure that the right number of people, with the right skills, is at the right place at the right time to deliver health services for the population needs, at an affordable cost [1–4].

In response of this, many initiatives have been created to train health workers and strengthen the health system [5–8]. In the Economic Community of West African States (ECOWAS) states members, the West African Health Organization (WAHO) aims to scale up the region's human resource base by promoting inter-divisional training, promoting training in specific technical areas, promoting cross-border skill transfer, spearheading the effort to ensure equivalency of degrees, curricula harmonization, ensuring placement of trainees, developing sub-regional centres of competence and excellence [9].

WAHO recognized the need within the West Africa sub-region for bilingual professionals who are skilled in public health, management, leadership, and information technology to build human capacity in public health. As a result, the Division of Human Resource Development of WAHO has developed the Young Professionals Internship Program (YPIP) in 2005 with his partner USAID in recognition of the lack of adequate human resource for health in the West African sub-region. It aims to produce young professionals from the ECOWAS sub-region who are able to demonstrate competencies in core technical areas that are crucial to the development of human resource needs for public health; to improve institutional capacity and mentoring capacity in participating regional organizations and institutions. This article explores the evolution of the YPIP and reports observations drawn from the first year of the eight-year programme.

### Project evaluation

The paper aimed at drawing out inferences from the literature and a strategy to develop health professionals in West Africa. We collected secondary data by accessing WAHO archives of intern biographical data, course curricula, intern selection data, socio-demographic data, intern qualifications and experiences, program logistical data, and other administrative/ management data. We also collected secondary data from an external independent evaluation of the programme recommended by WAHO from 2005 to 2010. The following tools were developed for data collection: anonymous online questionnaire intended for all past interns of the program, a semi-structured, key informant interview for 16 interns selected through purposive sampling, a self-administered questionnaire to employers and / or supervisors of key informant interns, a self-administered questionnaire to trainers of YPIP components, a semi-structured, key informant interview for the WAHO management team, a data collection matrix that house secondary information from WAHO archives. Due to the bilingual nature of the evaluation, all data collection tools were developed in both English and French. Data collection was carried out by administering the evaluation tools to select target groups. In some cases, the evaluation team conducted interviews in person, via telephone, or via internet. They gave questionnaires to self-administer and respond via e-mail. In other cases, data were collected online through the form of an online survey. We processed our data with Excel 2007 software.

### Structure of the intervention

WAHO is a specialized agency of ECOWAS with headquarters in Bobo-Dioulasso, Burkina Faso. YPIP is a 12 months programme starting in January which consists of 6 phases. The first stage (6 weeks) would cover introduction of interns to the program, training in core general areas including basic management skills, principles of public health, language and Computer and information technology skills.

The second stage (16 weeks) is the first placement in a host institution. During this period, interns, under the guidance of mentors, would be expected to acquire practical experience and special technical skills relevant to the interns’ areas of interest in health sector, to participate in problem solving in these institutions. The health priority technical areas for WAHO are HIV/AIDS, Reproductive Health, Child survival, Nutrition, Prevention of Blindness, Malaria, Health Research, and Disease Control.

The third stage (2 weeks) is a placement in ECOWAS Ministries of Health. This would be a study tour under the guidance of the WAHO Focal Points/Liaison Officers in the countries. Interns are expected to observe the functioning of ministry; to gain knowledge on the process of formulation and implementation of National health programmes and policies. The fourth stage (4 weeks) is a mid-term review at WAHO. During this stage, interns are expected to assess personal progress toward the acquisition of the desired competencies of the first placement and the study tour of Ministries; to use their experiences as case studies in improving problem solving skills; to acquire additional knowledge and skills in the core general areas of the program; to prepare for the second placement at Host Institutions. The fifth stage (18 weeks) is a second placement in the same host institution. This is similar to the first placement. Interns continue the learning experience; utilize the additional approaches, methodology, tools acquired during the mid-term review. Interns could be sent to another institution, if the mid-term review reveals that the situation at the Host Institution is not conducive for the attainment of the programmes’ objectives or there is a consensus for change of placement between the Mentor and the Intern.

The sixth stage (3 weeks) is a final Review at WAHO. During this final debriefing stage, Interns would be expected to assess progress accomplished toward acquisition of desired competencies, exchange experiences, evaluate the program, acquire knowledge and skills relevant to job search.

### Management

The YPIP programme is under the Division of Human Resource Development of WAHO. The first manager is the director who has mandated a supervisor, a coordinator and a secretary for the day by day management. The management staffs works with public and private partners, national and international programs and institutions, Non Governmental Organizations, WAHO focal point at the ministries of health of each West African country, and consultants.

### Selection process

The requirements for selection of trainees are based upon a set of pre-defined criteria focusing mainly on the experience and qualifications of candidates. Additionally, candidates face age and work experience restrictions to ensure that young and inexperienced candidates with potential get the opportunity to participate. An application for admission to the program is advertised on website www.wahooas.org and in all member countries and will provide:

- A letter of application addressed to the director indicating that the candidate wants to apply for the program
- A curriculum vitae comprehensively describing their professional experience(s)
- Official identification (e.g., passport, national identification, or birth certificate) certifying that the candidate is from a member ECOWAS country
- Certified copies of all diplomas and certificates
- A cover letter indicating the candidate′s reasons for seeking admission to the program, areas of expertise, and career plans
- Three letters of references, of which two must represent an academic affiliation and the third from the workplace

In addition, applicants are required to have the following competencies:

- Ability to speak and write at least one of the official languages of ECOWAS (English, French and/or Portuguese).
- Possession of a degree or vocational training with substantial experience in health
- Possession of basic computers skills

A four-phased approach is used to evaluate applications for admission.

**Phase One:** consists of a basic review of all the required components of the application. Applications received after the closing date and that do not include the minimum required components will be eliminated at this phase:

The minimum requirements of phase one includes:

1. Completed documents in accordance with the advertisement: letter of application, CV, photocopy National Passport or National Identity Card or Birth certificate, photocopies of certificates, letter of motivation, three Letters of Reference (at least two available)
2. University level or an equivalent professional qualification in health at least 3 years after the diploma of the secondary school
3. Citizen status within West Africa
4. Qualification dates not to exceed five years from the time of the submission of the application

**Phase Two:** consists of the elimination of candidates with inadequate requirements. After being assured that applications meet the minimum required criteria in Phase One, applicants are assessed against the rubric of the Table 1.

**Table 1:** Criteria and scores to evaluate requirements of candidates

No.	Item	Condition/Qualification	Score
**1**.	**University degree or professional qualification**	Anglophone: B.A., B.Sc, MBBS, MD, SRN/SRM Francophone: BAC + 3years	5 (max.)
**2**.	**Additional University or Professional qualifications**	<1 year >1 year but <2years >2 years	0.5 1 2 (max.)
**3**.	**Professional experience**	-Related to Health – note 1/an. - Not-related to Health	0.5 – 5 (max)
**4**.	**Employment status**	-Public Employment -Private sector -Unemployed	2 (max.) 1 0
**5**.	**Second official ECOWAS language**	-Certified (Authenticated) Claim without certification Beginner or no knowledge	2 (max.) 1 0
**6**.	**Competence in Data processing and Information Technology**	-Certified (Authenticated) Claim without certification Beginner or no knowledge	2 (max.) 1 0
**7**.	**Sex**	Female Male	1 (max.) 0
**Maximum score obtainable**			**20**

**Phase Three:** consists of a review of the quality of candidate requirements by reading and reviewing letters of motivation and more in-depth reviews of candidate profiles. A final shortlist is determined at the end of this phase.

**Phase Four:** consists of the final selection of candidates from the shortlist developed.

### Selected interns and beginning

After compiling and screening all application, selected interns results are posted on the website. A letter of offer to selected interns is sent them with further information.

- Each intern is entitled to a laptop at the beginning of the training. the laptop becomes the property of intern if he/she successfully completes the program
- Each francophone intern will be placed in a host institution in an Anglophone West African country and vice versa for the Anglophone intern. The Portuguese intern will have to choose between English and French as the language to be learnt. The intern cannot go back to his country until the end of the training except it becomes absolutely necessary.
- WAHO would pay the airfares and other transportation costs from headquarters in Bobo-Dioulasso to and from the host institution.
- Transport cost during the course of working in the institution should be borne by the institution. The intern would pay for his/her transportation to and from work from his/her monthly allowance.
- Host institutions should provide accommodation free of charge to the intern as much as possible. Where this is not possible, the institution should assist the intern to secure a reasonably prized accommodation.
- An allowance is given to the interns per month
- The intern has a medical insurance during the program

### Host institution

In 2012, WAHO worked with institutions across to field sites to host interns for the practical field experience. To facilitate the intern's host institution experience, WAHO organizes an information session early within the program to discuss related policy issues. The Table 2 shows that interns are posted in different institutions specialized in specifics domains. All the institutions implement health programs in Africa and have institutional interventions which align with intern interests.

**Table 2:** List of host institutions, locations and domains of the young professionals in 2012

Institutions	Locations	Domains
**National Malaria Control Program**	Abuja, Nigeria	Malaria
**EHORECON**	Abuja, Nigeria	Disease Control
**National AIDS Control Program**	Accra, Ghana	HIV/AIDS
**Ghana Health Services**	Accra, Ghana	Nutrition, Child & Reproductive Health
**National Nutrition Agency**	Banjul, The Gambia	Nutrition, Child Survival
**Medical Research Council**	Banjul, The Gambia	Health Research, Disease Control
**Centre Muraz**	Bobo-Dioulasso, Burkina Faso	Health Research, Disease Control
**Helen Keller International**	Conakry, Guinea	Nutrition
**Helen Keller International**	Dakar, Senegal	Nutrition
**Intra Health**	Dakar, Senegal	Reproductive Health
**Helen Keller International**	Freetown, Sierra Leone	Nutrition, Child Survival
**National AIDS Control Program**	Monrovia, Liberia	HIV/AIDS
**Helen Keller International**	Ouagadougou, Sierra Leone	Blindness Prevention
**JHPIEGO**	Ouagadougou, Burkina Faso	Malaria

### Number of beneficiary

**Figure 1:**
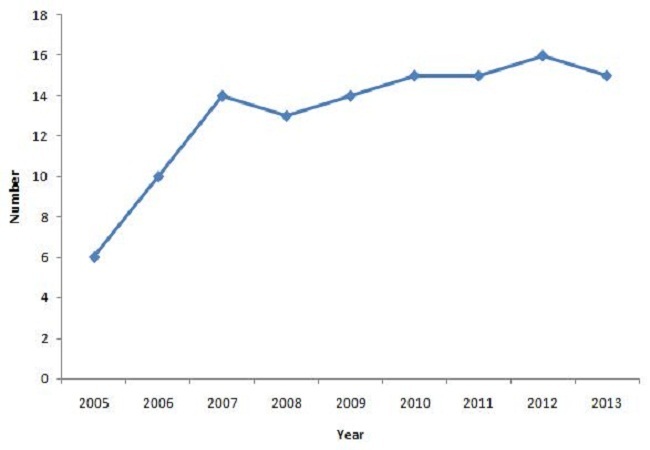
Evolution of the number of participants from 2005 to 2013

Since the 1^st^ batch in 2005, 103 young professionals participated to the programme from 7790 applications across 15 countries in West Africa (Benin, Burkina Faso, Cabo Verde, Ivory Coast, Ghana, Guinea, Guinea Bissau, Liberia, Mali, Niger, Nigeria, Senegal, Sierra Leone, The Gambia, and Togo). The percentage of selection of candidates was 0.18 (6/320) in 2005 and 0,01 (16/1587) in 2012. The Figure 1 draws an evolution of the participants and Table 3 shows that Nigeria and Ghana have the highest number of participation and Cabo Verde the lowest.

**Table 3:** Number of beneficiary per year and per country

COUNTRIES	2005	2006	2007	2008	2009	2010	2011	2012	2013	Total
**Benin**	1	1	1	1	1	1	1	1	1	9
**Burkina Faso**	1	0	1	1	1	1	1	1	1	8
**Cabo Verde**	0	0	0	0	0	0	0	1	1	2
**Ivory Coast**	0	1	2	1	1	1	1	2	1	10
**Ghana**	2	2	2	2	1	1	1	1	1	13
**Guinea**	0	1	1	1	1	1	1	1	1	8
**Guinea Bissau**	0	0	0	1	1	0	1	0	1	4
**Liberia**	0	0	0	0	1	1	1	1	1	5
**Mali**	0	1	1	1	1	1	1	1	1	8
**Niger**	1	1	1	1	1	1	1	1	1	9
**Nigeria**	0	0	3	2	1	2	2	2	1	13
**Senegal**	1	1	1	1	1	1	1	1	1	9
**Sierra Leone**	0	0	0	0	1	1	1	1	1	5
**The Gambia**	0	1	0	1	1	1	1	1	1	7
**Togo**	0	1	1	0	1	2	1	1	1	8
**Total**	6	10	14	13	14	15	15	16	15	118

### Courses

The main components taught during the programme are language, public health, management and leadership, information technology and communication.

Language Training: The goal of this component is to increase the capacity of learners in oral communication, particularly in areas in which the intern work and have a need. Specific objectives of the language training include building vocabulary in general and in specific areas, building grammar skills which are applied to conversations, building confidence and fluency to make communication easier, and building vocabulary in specific public health areas. The mastery of a second ECOWAS official language is a critical component of the YPIP program.

Public Health: This component provides interns with a basic background in public health to understand the major health problems of West African. The component also helps interns to appreciate effective and efficient programs that improve health status of ECOWAS citizens. The course is taught through a combination of methods of instruction suitable for adult learning, such as lectures and discussions, guided field visits, case studies, individual and group assignments. Topics include health system, health and disease, primary health care, epidemiology and importance of disease control of diseases, reproductive health and family planning, child health and child survival strategies, environmental health, health education and information systems, millennium development goals, biostatistics, health research, Software Epiinfo.

Management and Leadership: This component encompasses personal and organizational management, leadership, team work, negotiation, problem solving, strategic planning, partnering and advocacy, software MS Project.

Information Technology and Communication: This component introduces the fundamentals working with a Windows program, optimizing and maintaining a computer, networking with Windows, managing a workbook, using e-mail, health mapping and other advanced features.

### Current careers of interns

**Figure 2:**
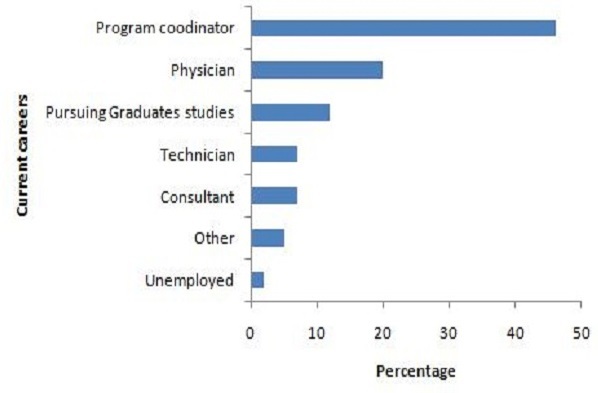
Distribution of the current careers of 41 interns from 2005 to 2010

Amongst 41 young professionals from 2005 to 2010, the places of their job after the programme were 66% National; 14% Sub-regional within ECOWAS; 10 > % International; and 10% Not Applicable due to unemployment, consultancy, and/or graduate studies. The Figure 2 draws that 46% of them have a coordinator position.

## Discussion

A review of the programs suggests that the YPIP has substantial potential to contribute to strengthened health systems, which, in turn, will help achieve WAHO objectives. However, the initiative faces a number of challenges in achieving these outcomes and demonstrating its success. Two major challenges will be a lack of focus and support on career and job placement directly after the programme, efforts to maintain ongoing communication and relationships with YPIP graduates. Despite these challenges, a number of lessons are already emerging that will contribute to the general knowledge and health systems capacity building.

There is a need to diversify and increase the number of host institutions for trainees in the various countries, as some interns have not been placed at compatible institutions. WAHO should formalize its collaboration with institutions and assess institutions’ commitment to provide adequate supervision for trainees. It should also establish a sustained dialogue with interns to choose institutions best suited for them. Additionally, it should foster stronger relationships between host institution mentors and interns so that they can work together to coach and mentor interns. Establishing an educational research framework provides a vehicle to discern evidence of good practice in the training of health care workers [10]. The management team should objectively investigate and address concerns related to stipends, insurance, and host institution logistical issues.

The commitment to develop young professionals comes with the responsibility to offer career guidance or provide job placement upon graduation from the program, especially if interns have left job posts that are no longer available upon completion of the program. Given the shortage of qualified human resources in the countries of ECOWAS, WAHO could consider placing interns at government agencies in their respective countries and avoid brain drain [4, 11]. Some promising students might also benefit from continued training with WAHO's sponsorship. To combat brain drain, trainings have been created in which healthcare professionals from Africa conducted the bulk of their research in their own countries; some are partly successful [12].

The Young Professional Internship Programme currently has low visibility, even within ECOWAS countries. More visibility is necessary to attract more applicants and increase the notoriety of the program from the perspective of employers and partner institutions. There is no form of structured interaction among alumni at this time, even though respondents are demonstrated an informal interest in maintaining an alumni network. WAHO could increase visibility, gain mentors, expand host institutions, and promote peer-to-peer job placement through the development of a formal alumni network. Restructuring the program to incorporate other sectors and disciplines related to health will help YPIP better respond to ECOWAS countries’ high demand for human resources. Increase number of interns per year because West African countries have a high demand for skilled professionals in priority areas and could benefit from additional YPIP graduates.

The strengths of the programme are a full time Coordinator who looks after all program components as a whole, sub-regional exposure with cross-cultural exchange, strong theories and rich curriculum, acquisition of a computer laptop and IT skills, acquisition of second language and bilingualism, opportunities to become more professional with practical field and work experience for interns out of their respective home countries (host institution), more and more applicants which can be seen as strength for the program.

## Conclusion

YPIP program has successfully carried out its original aims and objectives to equip young professionals with basic principles of public health, management, and leadership, acquire competence in a second official language (French, English, and Portuguese), information and communication technology. Contributing factors towards this successful evaluation included positive ratings and commentary from previous interns about the relevance, usefulness, and quality of the programme, encouraging feedback from WAHO management, trainers, administrators, and intern employers on the impact of the YPIP program on young professionals, supporting evidence that demonstrates increased knowledge in professional skills and language competency.
